# Self-Calibration Algorithm for a Pressure Sensor with a Real-Time Approach Based on an Artificial Neural Network

**DOI:** 10.3390/s18082561

**Published:** 2018-08-05

**Authors:** Ahmed M. M. Almassri, Wan Zuha Wan Hasan, Siti Anom Ahmad, Suhaidi Shafie, Chikamune Wada, Keiichi Horio

**Affiliations:** 1 Graduate School of Life Science and Systems Engineering, Kyushu Institute of Technology, 2–4 Hibikino, Wakamatsu-ku, Kitakyushu 808-0196, Japan; horio@brain.kyutech.ac.jp; 2 Department of Electrical and Electronic Engineering, Faculty of Engineering, Universiti Putra Malaysia, Serdang 43400, Selangor, Malaysia; sanom@upm.edu.my (S.A.A.); suhaidi@upm.edu.my (S.S.); 3 Institute of Advanced Technology, Universiti Putra Malaysia, Serdang 43400, Selangor, Malaysia; 4 Malaysian Research Institute on Ageing, Universiti Putra Malaysia, Serdang 43400, Selangor, Malaysia

**Keywords:** pressure sensors, self-calibration algorithm, artificial neural network, pressure measurement system, real-time application, robotic hand glove, rehabilitation applications

## Abstract

This paper presents a novel approach to predicting self-calibration in a pressure sensor using a proposed Levenberg Marquardt Back Propagation Artificial Neural Network (LMBP-ANN) model. The self-calibration algorithm should be able to fix major problems in the pressure sensor such as hysteresis, variation in gain and lack of linearity with high accuracy. The traditional calibration process for this kind of sensor is a time-consuming task because it is usually done through manual and repetitive identification. Furthermore, a traditional computational method is inadequate for solving the problem since it is extremely difficult to resolve the mathematical formula among multiple confounding pressure variables. Accordingly, this paper describes a new self-calibration methodology for nonlinear pressure sensors based on an LMBP-ANN model. The proposed method was achieved using a collected dataset from pressure sensors in real time. The load cell will be used as a reference for measuring the applied force. The proposed method was validated by comparing the output pressure of the trained network with the experimental target pressure (reference). This paper also shows that the proposed model exhibited a remarkable performance than traditional methods with a max mean square error of 0.17325 and an R-value over 0.99 for the total response of training, testing and validation. To verify the proposed model’s capability to build a self-calibration algorithm, the model was tested using an untrained input data set. As a result, the proposed LMBP-ANN model for self-calibration purposes is able to successfully predict the desired pressure over time, even the uncertain behaviour of the pressure sensors due to its material creep. This means that the proposed model overcomes the problems of hysteresis, variation in gain and lack of linearity over time. In return, this can be used to enhance the durability of the grasping mechanism, leading to a more robust and secure grasp for paralyzed hands. Furthermore, the exposed analysis approach in this paper can be a useful methodology for the user to evaluate the performance of any measurement system in a real-time environment.

## 1. Introduction

Pressure sensors are essential elements in many modern robotic applications, like robotic hands, due to their ability to facilitate data acquisition (DAQ) and to develop an actual measurement system. Day by day, robotic hands are becoming more important features in our daily life, as it involves the environment, grasping objects, performing tasks or even emulating the human hand. Some types of robotic hands have been implemented for various purposes such as dexterous manipulation [1,2], artificial limbs [3], grasping objects [4,5], rehabilitation applications [6,7,8] and pick and place applications [9]. However, in rehabilitation applications, the development of wearable robotic hand gloves based on pressure sensors for a measurement system involves the design of an adequate model with the least possible calibration time and highly accurate measurement data. Furthermore, it should be able to address different input sensors [10,11] and at the same time be able to overcome the nonlinearity output signal issue. In fact, the linearization of output signal sensors and the calibration process are the major items that are involved in defining the features of a pressure sensor.

In the literature, traditional methods to analyse and model output signal sensors have been introduced. For example, second-order models have been used in [12]. In these models, various techniques can be used to conduct more accurate results in linearization, such as analogue, digital and computer look-up ROM tables [13,14,15,16,17]. Other work has used metal oxide semiconductor (MOS) technologies to solve the nonlinear response. The aforementioned traditional calibration methods is a time-consuming task because calibration is usually done by manual and repetitive identification [18]. Moreover, the algorithm can be applied specifically for a particular sensor but is not appropriate to be used in a general measurement system.

Thus, as time elapses during manipulation in real time (RT), some pressure sensor parameters are changed due to hysteresis, variation in gain and lack of linearity [10,19]. This negatively affects the calibrated output data. Thereby, inaccurate pressure measurements appear. This implies that these sensors compensate to eliminate the systematic errors and to ease the calibration. Previous calibration algorithms for intelligent sensors have been implemented [11,20,21,22,23,24]. In detail, researchers have different options for algorithms, some of which are recursive algorithms [24,25] or artificial neural networks [10,26]. The former cannot be applied in a general way, one of which is the progressive polynomial algorithm [24]. Neither its effectiveness, nor the number of readjustment points required to achieve a minimal error have yet been proven. Furthermore, the calibration cost is still high. 

However, one study investigated the same proposed pressure sensor that was tested herein [27]. A nonlinear model based on traditional computational methods was proposed. Unfortunately, this model couldn’t be applied in real time when a high measurement accuracy for multiple sensors is needed. The mathematical relationship, through data interpolation using a traditional computational method, is inadequate for solving the problem. Since the pressure change of the sensor over time is one of the compounding variables, it is extremely difficult to resolve the mathematical formula because of the requirement of complex calibration, calculation procedures and limited improvement in accuracy [28]. In this case, a self-calibration algorithm based on artificial neural network is recommended to overcome the aforementioned issues.

Currently, neural networks are used for linearization in which the transfer function response curve of the sensor can be identified and the amplifiers linearized [29,30]. ANNs are advantageous because feedforward networks are universal approximators capable of learning continuous functions with any desired degree of accuracy [31]. In most cases, the ANN model was trained using the back-propagation (BP) algorithm [32]. ANNs offer a reliable tool that can model and predict complex problems [33]. It has the ability to capture complex interactions between different variables with a high self-learning capability. Furthermore, ANNs can be applied to a variety of tasks and problems, such as classification, interpretation, diagnosis, modelling, and control. They are more suitable to problems that are highly complex to solve by mathematical modelling or other classical procedures [32].

Therefore, based on the aforementioned advantages of ANN, this paper presents a novel approach to predicting the self-calibration of a pressure sensor with real time based on the Levenberg Marquardt Back Propagation Artificial Neural Network (LMBP-ANN) model. Our approach provides a self-calibration method to obtain and calibrate the model for pressure estimation to the grasped object using wearable robotic hand glove. After obtaining an accurate pressure for the grasped object, the secure grasp as well as rehabilitation system for patient with paralyzed hand can be successfully designed. 

Furthermore, the proposed model can simplify the self-calibration process by increasing the accuracy and reducing the time of training and evaluation algorithm. As a consequence, the confidence performance of the system is enhanced, and the cost of the calibration services is reduced. To validate the proposed approach, we provide real experiments of the pressure sensor with a comparison between the reference (obtained by the load cell) and the developed approach by using the LMBP-ANN model.

The paper structure is organized as follows: the measurement system consideration and calibration as well as ANN architectures and parameters are presented in Section 2; Section 3 presents the results of calibration pressure sensor, training and evaluation of the algorithm and the performance of LMBP-ANN model; the testing and evaluation of the trained network is shown in Section 4; and finally, Section 5 shows the main conclusions and future work.

## 2. Methods 

This section demonstrates the consideration of the measurement system including the conditioning and calibration of the pressure sensor used. Furthermore, it describes the artificial neural network model to be implemented for the self-calibration of pressure measurement system. The parameters of training algorithm to be applied are also introduced.

### 2.1. Measurement System Consideration and Calibration

**Figure 1:**
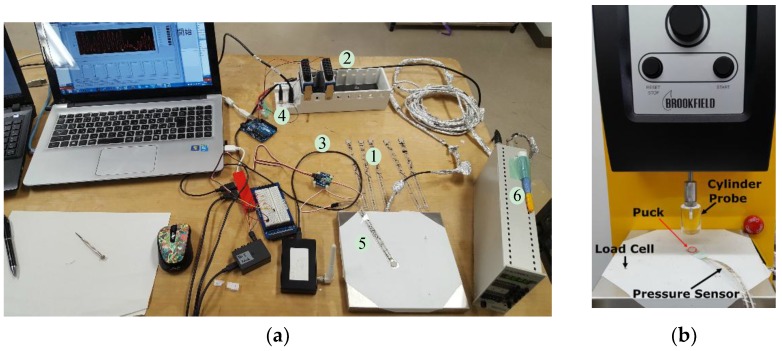
(**a**) The experimental setup conditioning of the FlexiForce sensor for the measurement system; (**b**) The CT3 Texture Analyser for evaluation of the pressure sensor.

The calibration process is being performed in real time or for dynamic manipulation in which a nonlinearity of the pressure sensors in the dynamic behaviour has occurred. Due to the calibration process, to ensure the high accuracy of the measurement system and optimal performance, the conditioning of the FlexiForce sensor (Tekscan, South Boston, MA, USA) model A201 has been utilized in dynamic loading. Figure 1a describes the experimental setup conditioning the FlexiForce sensor for the measurement system. Five FlexiForce sensors ① have been developed, and their characterization is defined, as well. In addition, the data acquisition device (DAQ) ②, conditioning circuit ③, trigger circuit using Arduino ④, load cell ⑤ and strain amplifier ⑥ have been used and incorporated into the system, as illustrated in Figure 1a. It is shown that in practical cases, the sensor is incorporated into a circuit to perform a pressure to voltage conversion. 

Before using the sensor, it was conditioned by applying 110% (or more) of the maximum test load onto the sensor for a few minutes to break it in. This process should be repeated various times in order to stabilize the output resistance to offer the best result. In this research, a dynamic application is one of the purposes to be implemented so that the performance of the pressure sensor is evaluated in a dynamic environment. 

Accordingly, a dynamic loading on the sensing area of the FlexiForce sensor was applied using the CT3 Texture Analyser machine (Tekscan, South Boston, USA) as demonstrated in Figure 1b. The machine performs the experiment by applying controlled forces in compression using a probe. In compression mode, a probe moves down slowly at the pre-test speed until a threshold value (the trigger) is reached. The probe then moves a set distance at a set speed into the sample pressure sensor that is placed on the base table of the load cell. The load cell will be used as a reference for measuring the applied force until the probe again returns to its starting position. The reference force was measured by a calibrated industrial load cell force sensor connected to a signal amplifier. 

A FlexiForce sensor A201 was used with a standard force range of 0–25 lb (110 N), sensing area of 0.375″ in diameter (9.53 mm) and 0.008″ in thickness (0.208 mm). The sensor consisted of two layers of substrate. This substrate is composed of polyester film that enhances the force sensing and improves the performance including linearity, hysteresis, drift and temperature sensitivity compared to any other thin film [34,35,36,37,38]. Furthermore, it is flexible and ultrathin enough so that the researchers and designers can use it in different integrated applications, as well as for applications that are oriented towards manipulative tasks based on wearable robotic hand glove. 

However, one of the aims of using the proposed measurement system is to create secure grasp for paralyzed patients by developing a wearable robotic hand glove with a robust sensing mechanism. Therefore, to ensure the highest performance and accuracy of the calibration process, a conditioning sensor was utilized by loading it at more than 30 N for a few cycles or pulses. The maximum load was selected to be an upper value of 44.13 N, as recent experiments have found that the typical maximum finger force produced by humans is no higher than 30 N [39]. In addition, a plastic puck (8.75 mm) in diameter is an object placed between the sensing area and load to ensure that the sensor captures 100% of the applied load if the contacting surface is larger than the sensor diameter and to reduce the high pressure for point load applications. 

The CT3 Texture Analyser with the standard cylinder probe made from plastic clear acrylic of 52.4 mm in diameter, 21 g and 35 mm long was used to apply a force on the sensing area of the pressure sensor. The diameter of the probe (52.4 mm) is larger than the sensing area of the sensor (9.53 mm) and the diameter of puck (8.75 mm) in order to imitate the real-world desired application and increase the performance output results. 

Generally, rehabilitation of the upper limbs is crucial for paralyzed patients; one hour of repetitive exercise every session is desired to recover the hand function, which is executed within three stages with 20 min for every stage. The target of this paper is to generate a secure grip for paralyzed patient by ensuring, and covering; the analysis of the pressure output signal within at least 20 min of dynamic manipulation as a first stage towards recovering hand functions. Thus, five FlexiForce sensors were used to conduct the experiment, which were later distributed to the five fingers of the wearable robotic hand glove. Subsequently, to evaluate how the sensor behaves over time, a dynamic calibration of 20 min was performed for each sensor with different maximum loading and holding times. 

In this paper, the first stage, using the CT3 Texture Analyser machine, was performed with a number of compression pulses upon a pressure sensor with 44.13 N and a 10 s holding time at the maximum load in every pulse and a 100 Hz sampling rate. The proposed test speed of the machine probe is 0.1 mm/s, which takes into consideration the sensor’s thickness (0.208 mm). 

The implementation and training algorithm for dynamic calibration is essential, and generation of a data set is important in order to create a potential model that is able to enhance the grasping mechanism. Thus, the dataset was collected, including the output voltage from the sensor and its corresponding target pressure. The proposed model is being implemented based on the Artificial Neural Network (ANN) using the dynamic calibration dataset. 

Prior to any ANN model design, some considerations were necessary. The output electrical signal x′ of any sensor in response to the input variable v′, which will be measured, is defined by:

(1)  x′ = f (v′)

In most of the cases, the input and output variables of pressure sensors have different scales, and they should be normalized in the range of [0, 1] to simplify their manipulation. This can be obtained by the normalization method as mentioned in [10]. In addition, the desired output signal is a straight line with unit slope, and this will be the target in the calibration process and will be the reference signal (t). Finally, the least mean square error (MSE) will be used to determine the linearity of the output signal (y) of the ANN:

(2) $$\varepsilon_{mse}=\frac{1}{N}\sum_{n=1}^{N}{\left(y_{n}-t_{n}\right)}^{2}\;$$

### 2.2. Artificial Neural Network Architectures and Parameters

**Figure 2:**
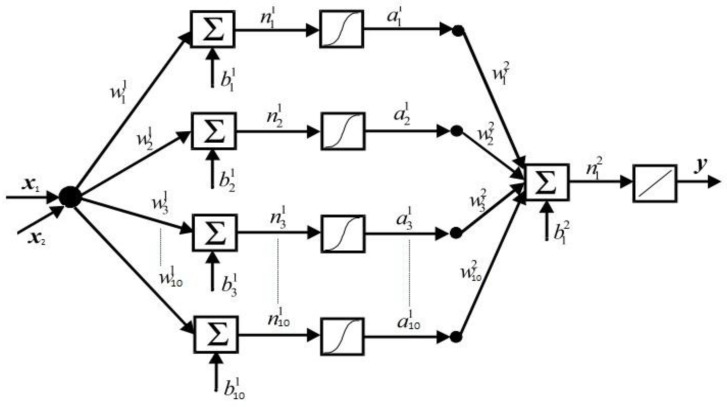
Architecture of the artificial neural networks used.

Since there is no commonly accepted optimal method to determine the best architecture of an ANN, a trial and error approach was adopted [32]. The number of neurons, number of layers, activation functions, training algorithm and the computation requirements are the major characteristics considered during the design. These features were determined under the restriction of archiving the least output error and the simplest ANN structure. Accordingly, the design of the network architecture started with fewer hidden neurons and then the number of hidden neurons was adjusted. The proposed architecture of the ANN model that provided best generalization was retained and is illustrated in Figure 2. In fact, multiple layers of neurons with nonlinear transfer functions allowed the network to learn the nonlinear and linear relationships between the input and output vectors. Therefore, the proposed network consists of two inputs (nodes) representing the parameters influencing the self-calibration of pressure sensor, along with 10 neurons in the hidden layer and a logarithmic activation function. The output layer is a single neuron with a linear activation function representing the pressure pattern as indication of self-calibration. 

The output of the ANN used is defined by:

(3) $$\;y=\mathrm{Purelin}\left[w_{i}^{2}\left(\mathrm{Tan}\mathrm{sig}\left(w_{i}^{1}x+b_{i}^{1}\right)\right)+b_{i}^{2}\right],\;i=1\;\mathrm{to}10\;$$

where x is the normalized output sensor signal, the weights are represented by the vector w, b is the bias and y is the linearized or self-calibration signal. 

There are several known activation functions including the sigmoid, ramp and Gaussian functions that allow neural networks to solve difficult problems. In the case of a multilayer network with receptive fields, using the sigmoid function as an activation function is generally recommended [40]. In this study, the tan-sigmoid function, shown in Equation (4), was used in the hidden layer and a pure linear transfer function, as shown in Equation (5), was used in the output layer neurons:

(4) $$\mathrm{Tansig}\left(x\right)=\frac{2}{1+e^{\left(-2x\right)}}$$

(5)  Purelin(x)=x

Several topologies of ANN were evaluated such as multiplayer perceptron (MLP) and radial basis function (RBF) [41,42]. Both types of networks are competent of universal approximation capabilities. Although, it is known that RBFs are worthy function approximation systems, the MLP was selected because is simpler than the RBF. Furthermore, the RBF network is computationally demanding [43,44]. However, a faster algorithm, by minimizing the MSE, increasing the convergence speed and selection of the appropriate learning rate, are the main roles of a successful training of the algorithm. The Levenberg-Marquardt backpropagation (LMBP) was designed for minimizing functions that are sums of squares of other nonlinear functions. Furthermore, it is able to reduce the sum of squares at each iteration [45]. The flowchart of this algorithm is clarified in [10]. This is very well suited to neural network training where the performance index is the MSE that is desired in this research. Consequently, for our proposal, the most appropriate ANN model to be implemented was a feed forward MLP based on LMBP training algorithm. 

Table 1 shows the specifications and values of the parameters used in the LMBP-ANN model. The calibrated data are randomly classified into training, validation and testing subsets to avoid any bias. Additionally, a traditional training-validation-test procedure is adopted for the optimization of an ANN model. The training set was used to build model structures and to determine the ANN weights and biases to minimize the error function and maximize accuracy in each iteration. The validation set was used to validate an optimal parameter set to avoid overfitting and provides an unbiased estimate of the generalization error of the model. Conversely, the testing set was only used to explore the performance of the trained model and confirm its generalizability. The training continued until the validation error failed to decrease for six iterations (validation stop).

However, the MATLAB program was used to execute the LMBP-ANN training process and output calculation based on the proposed method. The training process of the proposed network model updated the weight values in the connection between neurons. The outputs of the ANN used, as defined in Equation (3), were calculated in order to include the biases and weights. At each time an artificial neural network was trained, this could result in a different solution due to different initial weights and biases values and different divisions of data into training, validation and test sets. As a result, different artificial neural networks trained on the same problem could give different outputs for the same input. To ensure a high accuracy result of training ANN, it was retrained several times. 

Accordingly, a trial-and-error approach was used, and the calculated results of weights and biases are the best output obtained after several times of training the algorithm. The output of training was updated automatically in each iteration until it reached the highest performance by achieving the minimum MSE. This means a self-calibration method has been achieved and accomplished successfully.

## 3. Results

Here, we present the results of the proposed method. The analysis and experimental results for the developed measurement system using the pressure sensor are presented. After that, the training and evaluation of the self-calibration algorithm were implemented. Finally, the performance of the LMBP-ANN model was validated. 

### 3.1. Calibration Pressure Sensor

**Figure 3:**
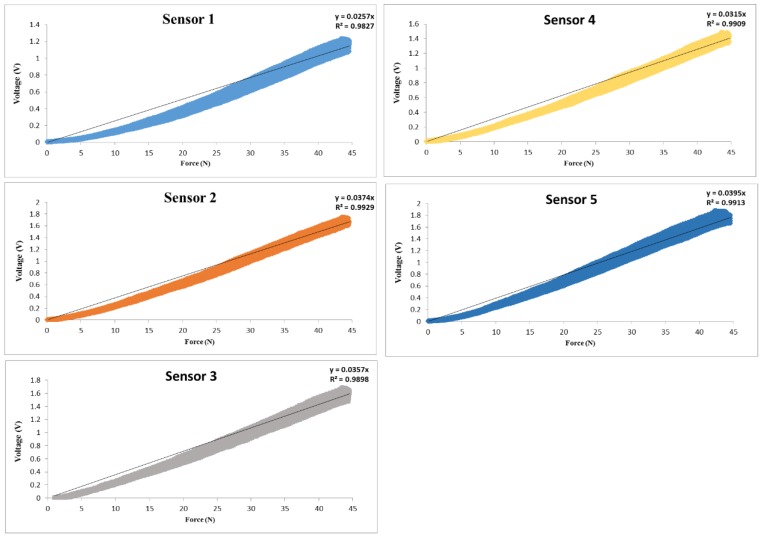
Dynamic calibration output voltage versus force of five pressure sensors.

The dynamic calibration process involved the calibration of the measurements as they changed over time. The CT3 Texture Analyser was used to apply continuous force on the pressure sensor. As the pressure changed with time, the type of calibration recommended was dynamic calibration. Thus, the calibration of the five pressure sensors was implemented dynamically. However, to make reasonable inferences for the application, such as the grasping mechanism analysis and the secure grasp using the wearable robotic hand glove, it was necessary to have an accurate measurement data from pressure sensors. Hence, the sensor signal model needed to be analysed to learn the fundamental relation between a sensor reading (V_o_) and an applied force (F). An analogue conditioning circuit based on the pressure measurement system was employed to calibrate and extract data from the utilized sensors to be processed. The fundamental relationship between a sensor reading, V_o_, and an applied force, F, was recorded for five pressure sensors, as described in Figure 3. The non-stationarity of the force sensor signals due to variation in its characterization was obvious. The equations of the relationship between the voltage and target pressure for each sensor were calculated, and these were used as a reference for further calibrations. Although the same sensors were utilized under the same experimental conditions, different measurement outputs were recorded. 

**Figure 4:**
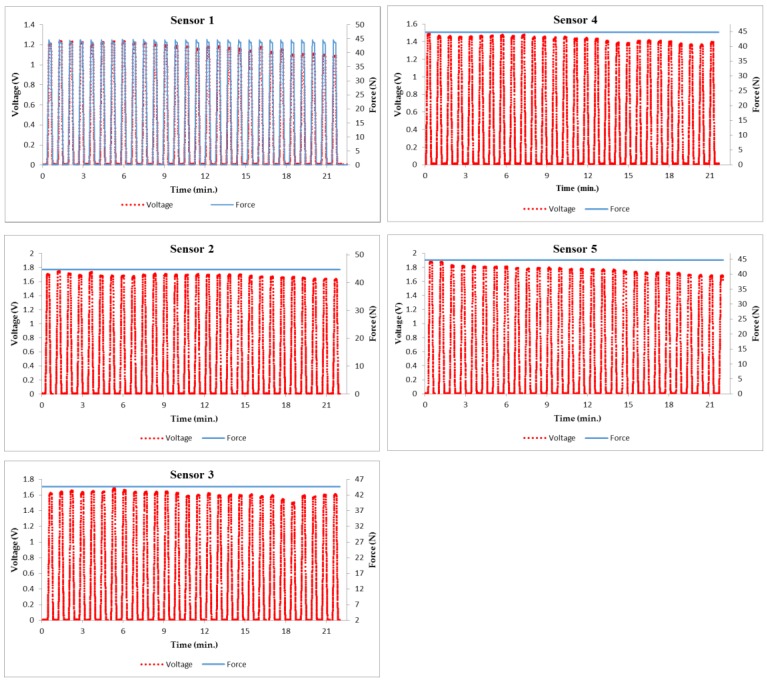
The relative measured voltage change in the sensor over the course of 20 min while applying a dynamic force of 44.13 N (28 repetition cycles, 10 s holding time, 100 Hz sample rate).

To additional clarify the behaviour of each sensor over time, the process of calibration was repeated several times, and the errors due to nonlinearity, hysteresis and non-repeatability were determined. Figure 4 demonstrates the relative measured voltage change of the sensor over the course of 20 min while exerting a dynamic force of 44.13 N with 28 repetition pulses and a 10 s holding time at the maximum load. Therefore, sensor 1 of Figure 4 clarified the behaviour of output voltage while applying a dynamic force for 20 min. A decrease in the output voltage over time was observed even as the same pressure forces were applied in each pulse. For instance, it decreased from 1.237357 V to 1.087904 V over the entirety of the calibration period. 

Moreover, as different sensors were utilized under the same conditions, different measurements of the relative output voltage were detected. For instance, the maximum output peak voltages for sensor 1 to sensor 5 during the whole period of calibration under the same applied pressure were 1.237357, 1.751546, 1.687251, 1.494452 and 1.883202 respectively. The changes in voltage output and the ultimate voltage of the sensor 5 were the highest due to material creep of piezoresistivity over time.

This means a symmetric error such as hysteresis, variation in gain and lack of linearity had appeared, which negatively affected the measurement systems. Thus, in order to develop pressure sensors for measurement systems in real time with improved features of calibration time and high accuracy, implementation of a self-calibration algorithm was recommended.

### 3.2. Training and Evaluation

In the proposed method, Levenberg Marquardt Back Propagation Artificial Neural Network (LMBP-ANN) is implemented through the MATLAB Neural Network toolbox with some modifications. A back propagation algorithm is commonly used to train feed forward networks. In the LMBP, the data flows from input layer to the hidden layer and finally to the output layer. The error signals generated in the output layer is back propagated to the hidden and input layers. The sum of squares error signals is minimized by adjusting synapse weight coefficients.

**Figure 5:**
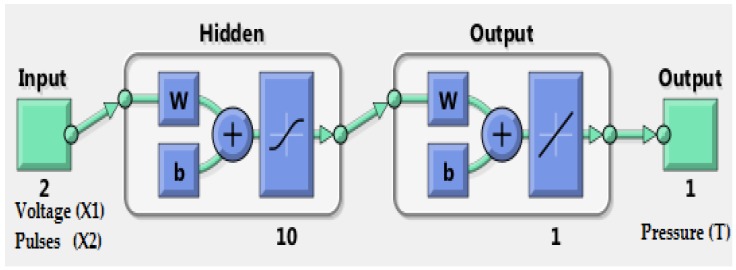
Two-layer feedforward network with a sigmoid transfer function in the hidden layer and a linear transfer function in the output layer.

For the training algorithm, the generation of data sets is important. The data were collected from the pressure measurement system using five pressure sensors and the CT3 Texture Analyser machine as well as load cell force sensor that was used as a reference for the calibrated data. The training ANN was performed for each pressure sensor with more than 132,000 calibration data obtained during the 20 min of dynamic loading. The topology of the proposed LMBP-ANN model is demonstrated in Figure 5. For each sensor of experimental calibration, 132,243 dataset samples were obtained while applying dynamic pressure using CT3 Texture Analyser machine. 

Since there was a decrease in the output voltage over time along with fluctuation, time was considered as an input of the ANN model and identified dynamically. Consequently, instead of using time, the number of cycles or pulses was selected because it can represent the decrease of output voltage during calibration process. 

Therefore, a 2 × 132,243 matrix of measurement data points from sensor 1 was identified for the algorithm to be trained. One row represented the output voltage of the pressure sensor, and the second one represented the number of cycles or pulses obtained along with the output voltage over time. The target output of the training algorithm was the pressure with a 1 × 132,243 matrix that was obtained from the reference of the load cell along the calibration process. Table 2 demonstrates the input and target data set of the training algorithm for pressure sensor 1. The table shows the calibrated data of voltage output sensor and its corresponding target pressure from the first pulse of calibration until the last one. The fluctuation in obtained voltage of the same pulse was measured and compared to other pulses. 

**Figure 6:**
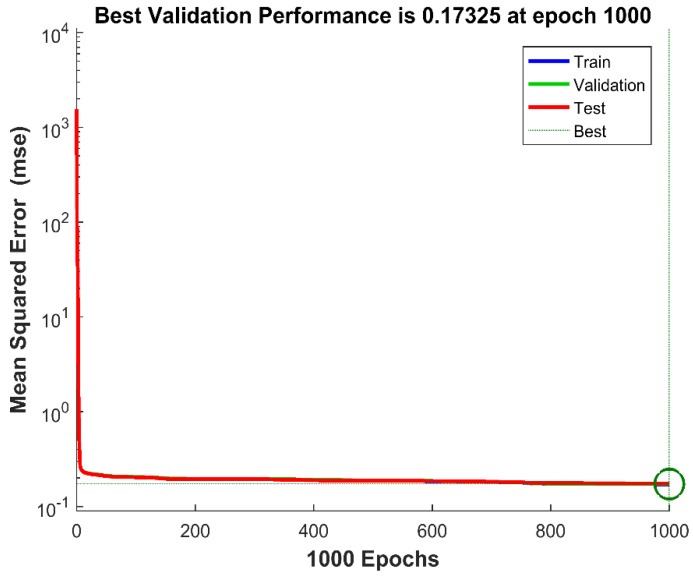
The performance of training, validation and test errors with training epochs.

The training process continued, epoch after epoch, until a best validated performance was found. Figure 6 shows variations of the MSE with training epochs. The errors of the training data, validation data and testing data kept decreasing until the validation error failed to decrease for six iterations (validation stop). In this training, the trained network had the best validated performance at epoch 1000. It was obvious that the result was reasonable because of the following considerations: the final MSE was small (0.17325); the test set error and the validation set error had similar characteristics, and no significant overfitting occurred by Iteration 1000 (where the best validation performance occurred).

### 3.3. Performance of the LMBP-ANN Model

**Figure 7:**
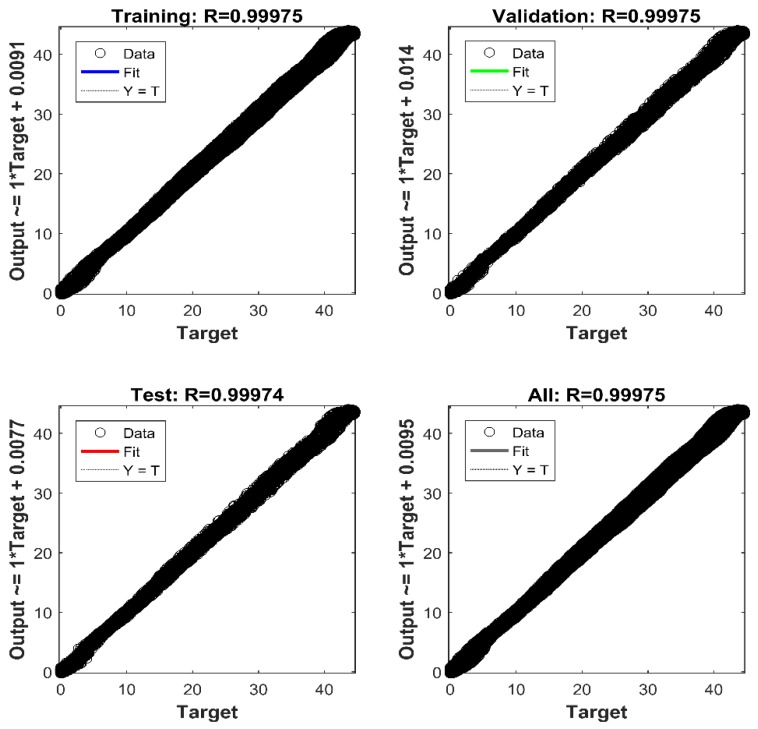
The agreement between the network’s outputs pressure and target pressure for training, validation, test and complete data set.

The performance of the LMBP-ANN model mainly depended on its ability to predict the experimental output data with reasonable accuracy. As shown in Figure 7, the ANN model output was able to accurately predict the self-calibration of the pressure relative to the actual experimental data. For instance, the coefficient of determination (R^2^) of model prediction versus experimental data for the training, validation and test data sets are 0.99975, 0.99975, and 0.99974, respectively. Thus, it can be argued that the proposed ANN model captured the relationships between the provided input and output data with acceptable accuracy, indicating excellent performance. Furthermore, regardless of the fluctuating input pressure data set to the trained network, a remarkable agreement between the network’s output pressure and target pressure for all data was observed.

The reliability of the proposed LMBP-ANN model for the complete data set was also evaluated via the mean-square error (MSE) and coefficient of determination (R^2^) between the model’s predictions and experimental results. The MSE value according to Equation (2) was 0.17325 and R^2^ value was 0.99975, which also indicated adequate performance. 

**Figure 8:**
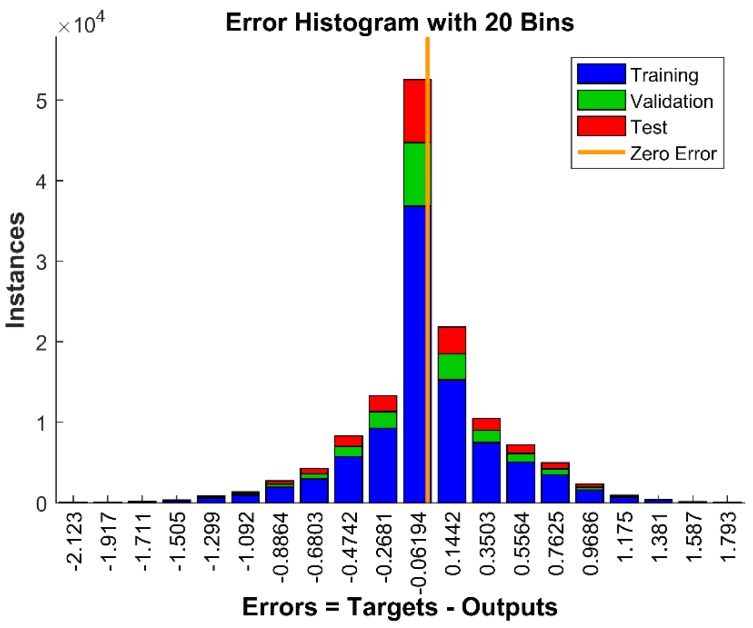
Structure of a 20-bin histogram using the LMBP training algorithm based on ANN.

In addition to the aforementioned validity of the network performance, the histogram method was also used to obtain additional verification of the network performance. Figure 8 demonstrates the data distribution of a continuous variable, and the blue, green and red bars represent training, validation and testing data, respectively. The histogram can give an indication of the outliers, which are data points where the fit is significantly worse than the majority of data. In this case, most of the errors fell between −1 and 1, and there were no outliers for either training or validation points, as evidenced in the testing regression plot. Hence, there was no need to collect more data, and the 20 min of calibrated data was enough for this training algorithm. 

The weight and bias matrices for the trained neural network at epoch 1000 are shown below, which were saved and will be programmed into the microcontroller in order to determine the pressure compensation. 

$$w^{1}=\left[\begin{array}{cccccccccc} 1.7726 & 3.4390 & -1.7749 & 10.6532 & 1.9292 & -1.9490 & -1.7341 & -2.3523 & 30.8504 & 0.1039\\ -10.5291 & 14.2303 & 10.8452 & 1.2110 & 0.1852 & -0.4000 & -0.1946 & 4.2049 & -0.1098 & 8.7989 \end{array}\right]$$

$$b^{1}=\left[\begin{array}{cccccccccc} -9.4999 & -12.0687 & 9.7591 & -7.7675 & -0.5226 & -1.8499 & -1.5887 & 5.1865 & 29.6176 & 10.0108 \end{array}\right]$$

$$w^{2}=\left[\begin{array}{cccccccccc} 1.0908 & 0.0067 & 1.0582\; & 0.0725\; & 0.3733 & 0.7917 & -1.6182 & 0.0377 & 0.0921 & -0.1490 \end{array}\right]$$

$$\;b^{2}=\left[-0.1844\right]$$

w^1^ represents the weight between the input layer and the hidden layer, w^2^ represents the weight between the hidden layer and the output layer, and b^1^ and b^2^ denote the bias of the hidden layer and the output layer, respectively.

## 4. Neural Network Testing and Evaluation with Untrained Data Sets

The performance and effectiveness of the trained network were further evaluated using a new independent input data set that the algorithm has never been trained on. Two new vectors, with 3600 input data, were introduced to the LMBP-ANN model in order to predict the self-calibration of pressure. One vector represents the input voltage and the other one denotes the number of pulses for pressure sensor 1 as a sample. Voltage data, with values from 0 to 1.2 in 0.01 increments, were fed into the neural network simultaneously with number of pulses. The matrix of 1 × 3600 was the output of the trained algorithm (predicted data), which represents the desired pressure. 

**Figure 9:**
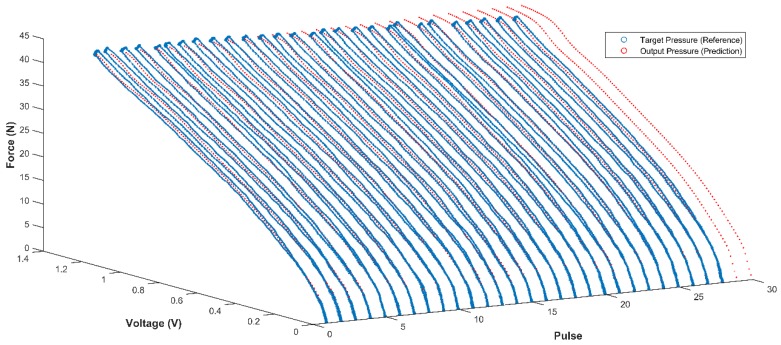
Output trained network performance versus the target (reference) for 20 min onward using untrained data set input.

As shown in Figure 9, the proposed LMBP-ANN model was able to predict the self-calibration of pressure relative to either with target pressure (reference) or without target pressure. It is clear that the predicted output tracked the target successfully during the first 28 pulses which were calibrated previously as introduced in Section 3.1. The estimated pressure pattern in the middle of target can be observed from Figure 9 and there are no data outliers. Furthermore, another two pulses after pulse 28 were predicted correctly even when no reference had been used. The results verify that the trained neural network can effectively correct and accurately measure the input pressure, which proves sufficient performance.

**Figure 10:**
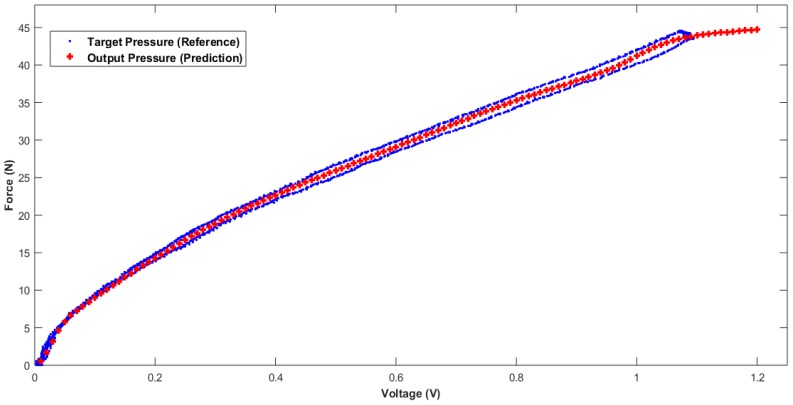
Output trained network performance versus target (reference) for pulse number 28 (20 min onward).

To clarify more how the proposed LMBP-ANN model performs either after 20 min of calibration time or if the applied pressure was higher than target pressure (reference), the model was tested from pulse 28 onward as shown in Figure 10. The blue curve is the target pressure, which represents the reference for the tested data, while the red is the output pressure (predicted) of the trained neural network for a new untrained input data. As appears in the graph, the proposed model has effectively estimated the desired data in line with the target and has achieved a typical performance at pulse number 28 (20 min onwards). After that, a new predicted data has been successfully estimated and it was compatible with the estimated pattern even the reference no longer exists, as shown at the end of curve. However, the two blue lines of target pressure are due to the hysteresis of sensor calibration. Although the hysteresis of the sensor had increased during the process time, the proposed model kept the prediction of the desired data within the target range. 

As a result, the proposed LMBP-ANN model for self-calibration purpose was able to successfully predict the desired pressure over time, even the uncertain behaviour of the pressure sensors due to its material creep. This means the proposed model overcomes the problems of hysteresis, variation in gain and lack of linearity over time. 

## 5. Conclusions

In this paper, a novel pressure sensor self-calibration estimation method is developed for a secure grasp of a paralyzed hand based on wearable robotic hand gloves. The developed method was implemented using the proposed Levenberg Marquardt Back Propagation Artificial Neural Network (LMBP-ANN) model. The LMBP training algorithm was effectively applied in the ANN model to minimize the MSE and to determine the optimal parameters that govern the input-output relationship of the model. Experimental data of pressure measurement system was collected in real time. With the pressure data obtained, the developed LMBP-ANN model was trained. 

The experimental results showed that the developed LMBP-ANN model can accurately estimate the applied pressure, and its performance is advantageous over other traditional methods with respect to estimation accuracy in real time. The reliability of the proposed model for the complete data set was evaluated via the mean-square error (MSE) and coefficient of determination (R^2^) between the model’s predictions and experimental results. The MSE value was 0.17325 and R^2^ value was 0.99975, which indicated adequate performance. Future works are related to the implementation of this method in microcontroller using wearable robotic hand glove based on pressure sensing mechanism.

## Figures and Tables

**Table 1:** The specifications and parameters of ANN model.

Training Parameters	Values
Neural network model used	Feed forward
Input nodes	2
Hidden layer	1
Hidden layer neurons	10
Output layer neurons	1
Output nodes	1
Training network algorithm	LMBP
Training percentage	70
Testing percentage	15
Validation percentage	15
Transfer function hidden layer	Tan-sigmoid
Transfer function output layer	Pure line
Data division	Random
No. of epochs	1000
Validation checks (iterations)	6
Performance	Mean squared error (MSE)

**Table 2:** The inputs and target data set used for neural network training.

Input		Target	Note
X1 (Voltage)	X2 (Pulses/Time)	T (Pressure)	Input (2 × 132,243) Target (1 × 132,243)
0.006557	1	0.181619	Start pulse no. 1
0.002981	1	0.1831097	
0.006472	1	0.1821192	
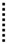	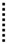	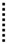	Continue until the end of pulse no. 1
0.005791	1	0.093957	
0.007238	1	0.096928	
0.005024	1	0.094349	
0.006046	2	0.085543	Start pulse no. 2
0.006472	2	0.072284	
0.005876	2	0.058114	
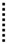	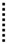	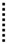	Continue until the middle of last pulse (no. 28)
1.080495	28	44.222411	
1.081602	28	44.216517	
1.085690	28	44.209515	
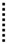	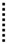	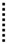	
0.005791	28	0.083052	This is the last row of 2 inputs and 1 output
0.011241	28	0.070892	
0.006472	28	0.057947	

## Supplementary Materials

The following are available online at http://www.mdpi.com/1424-8220/18/8/2561/s1, Video S1: Dynamic calibration of pressure sensor using the CT3 Texture Analyser while applying force of 44.13 N (10 s holding time, 100 Hz sample rate).
